# Instant Mercury Ion Detection in Industrial Waste Water with a Microchip Using Extended Gate Field-Effect Transistors and a Portable Device

**DOI:** 10.3390/s19092209

**Published:** 2019-05-13

**Authors:** Revathi Sukesan, Yi-Ting Chen, Suman Shahim, Shin-Li Wang, Indu Sarangadharan, Yu-Lin Wang

**Affiliations:** 1 Institute of Nanoengineering and Microsystems, National Tsing Hua University, Hsinchu 300, Taiwan; revu13@gmail.com (R.S.); et800619@yahoo.com.tw (Y.-T.C.); sumanshahim@gmail.com (S.S.); w0970711363@gmail.com (S.-L.W.); indu.4391@gmail.com (I.S.); 2 Department of Power Mechanical Engineering, National Tsing Hua University, Hsinchu 300, Taiwan

**Keywords:** field-effect transistors (FETs), ion selective membrane (ISM), extended gate devices, heavy metal ion detection, mercury

## Abstract

Mercury ion selective membrane (Hg-ISM) coated extended gate Field Effect transistors (ISM-FET) were used to manifest a novel methodology for ion-selective sensors based on FET’s, creating ultra-high sensitivity (−36 mV/log [Hg^2+^]) and outweighing ideal Nernst sensitivity limit (−29.58 mV/log [Hg^2+^]) for mercury ion. This highly enhanced sensitivity compared with the ion-selective electrode (ISE) (10^−7^ M) has reduced the limit of detection (10^−13^ M) of Hg^2+^ concentration’s magnitude to considerable orders irrespective of the pH of the test solution. Systematical investigation was carried out by modulating sensor design and bias voltage, revealing that higher sensitivity and a lower detection limit can be attained in an adequately stronger electric field. Our sensor has a limit of detection of 10^−13^ M which is two orders lower than Inductively Coupled Plasma Mass Spectrometry (ICP-MS), having a limit of detection of 10^−11^ M. The sensitivity and detection limit do not have axiomatic changes under the presence of high concentrations of interfering ions. The technology offers economic and consumer friendly water quality monitoring options intended for homes, offices and industries.

## 1. Introduction

Mercury or Hydragyrum is ubiquitous and has been used by mankind for generations in applications ranging from alchemy to thermometers, dental amalgams and even light bulbs [1]. Contamination of the biosphere by this toxic heavy metal has increased dramatically since the industrial revolution, for instance through coal burning and mining. The majority of the atmospheric mercury released by natural phenomena like volcanic eruptions and the above mentioned anthropogenic activities settles in the hydrosphere [2]. Mercury is proven to be a devastating neurotoxin with significant health consequences and diverse long term effects as a consequence of air inhalation, dental amalgams or the consumption of contaminated water [3,4]. Bio-accumulation of methyl-mercury in the food-chain of marine biota especially involving sea food consumers is an issue of potentially life-threatening neuro-developmental disorders [5]. It has been classified as a teratogenic element and falls under the top ten toxic heavy metals [6,7]. Once mercury enters the human tissue, it complexes with amino acids due to its affinity for sulfhydryl groups. It gets deposited in the human or fetal brain as it easily penetrates the blood brain barrier, proceeds through the placenta and even in adrenals, liver, kidney, pancreas, sweat glands, myocardium and testes to ultimately cause dysfunction [2]. 

The United States Environmental Protection Agency (EPA) has fixed the maximum contaminant guidance levels for Hg in drinking water to 2000 ng L^−1^ (9.97 × 10^−9^ M) or that of the aquatic life criteria as 1400 ng L^−1^ (6.98 × 10^−9^ M) (acute exposure) and 770 ng L^−1^ (3.50 × 10^−9^ M) (chronic exposure) [3]. The monitoring of mercury contamination levels is of high importance, especially in regions where sea food is consumed or where water contamination by heavy metals is prevalent. The need of the hour is inevitably to design and develop a robust, portable and cost effective sensor which can monitor the concentration of mercury ions in water and liquid foods. Bench top laboratory instruments presently at use include the atomic absorption spectroscopy (AAS), X-ray fluorescence (XRF), inductively coupled plasma-atomic emission spectroscopy (ICP-AES), inductively coupled plasma-atomic mass spectroscopy (ICP-MS), electrochemical and fluorescence techniques [8,9]. For the above mentioned bulk machinery, the detection limit is very low at close to parts per trillion (ppt). However, they do not find application as a convenient detection instrument for daily use as they require laboratory set-up, highly trained personnel and finance. The novel detection method employed in this work uses extended gate FET configuration with polymeric membrane as a substrate with target ionophores deposited on a gate sensing area. 

Previously, an AlGaN/GaN high electron mobility transistor (HEMT) sensor was used to detect heavy metal ions in water [10]. This structure employed an external gate electrode which is placed at a short distance (in the range of micrometers) away from the active channel of HEMT. The ion selective membrane is immobilized on the channel open region, leaving the gate open region free to be in contact with the solution [10]. In this work, on the sensing region, both reference and sensing electrodes are coated with ISM which diminishes the effect of solution conductivity, providing a higher sensitivity and a lower detection limit. Extended gate configuration employed in the sensor array provides less variation since a single FET with multiple gate sensing elements is used. In our sensor configuration, on application of positive gate bias, capacitive changes occur at the interfaces, leading to changes in the transistor drain current [11,12,13,14]. These features make it optimal for use in clinical studies, chemical contaminant sensing and environmental sample examinations. For a traditional Hg ISE, the limit of detection limit is 10^−8^ M [15,16] while we are able to detect lower concentrations of up to 10^−13^ M, eliminating the need for a reference electrode [17,18,19]. The field effect transistor has various advantages such as quick measurement, a simple electrical signal read out and elevated sensitivity [20,21,22]. In this research we have developed a device with a simple user interface and a very short response time (within five minutes) that can extrapolate the concentration of mercury ions in a blind test solution. Most importantly, ‘Sensitivity beyond the Nernst’ was investigated thoroughly by the “varying gap test”. Capacitance based modeling of the sensor is proposed which explains the efficiency of the membrane. A detection limit of 10^−13^ M was reported irrespective of the pH of the test solution with a life time of six months. Hence, our sensor can be used as a portable, cost-effective, daily-use gadget to test dynamic water quality.

## 2. Experimental

### 2.1. Structure of Extended Gate Hg-ISMFET

**Figure 1:**
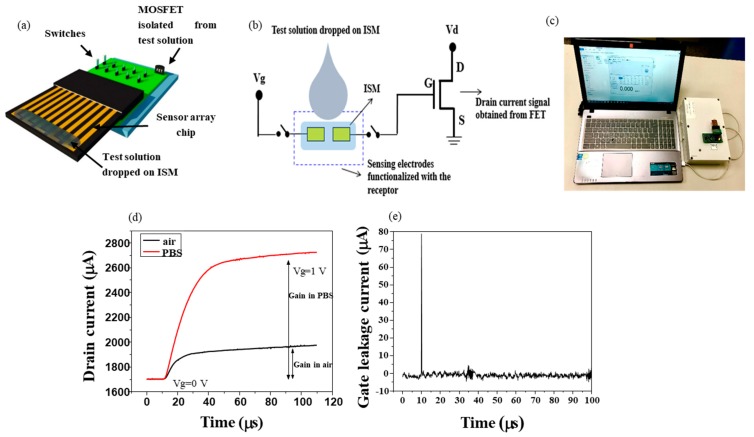
(**a**) Schematic representation of extended gate Hg-ISMFET connected to the prototype. (**b**) Structural representation of extended gate Hg-ISMFET. (**c**) Real view image of sensor chip mounted on the portable measurement system connected to personal computer. (**d**) Current gain average verses time in air and in 0.02X PBS by extended gate Hg-ISMFET. (**e**) Gain leakage current of ISMFET in the presence of 10^−8^ M mercury ions.

The schematic structure of the extended gate Hg-ISMFET is shown in Figure 1a. An extended gate FET sensor integrated with a poly vinyl chloride (PVC) based ion selective membrane is utilized. The sensor structure consists of a gold electrode array fabricated on a thermo-curable epoxy resin (Figure 1b). PDMS molds are made and the epoxy resin is poured onto it and treated for 1 hour at 125 °C and 1.5 hours at 165 °C respectively in oven. The epoxy resin is pulled off from the PDMS mold after curing and patterned for metal deposition using photolithography. Electron beam (e-beam) evaporator is used to deposit Ti (200 Å) and Au (2000 Å) on the epoxy substrate followed by lift off. By using 2 µm thickness SU-8 photoresist, the gold electrodes are passivated and sensing regions are opened by patterning using photolithography on the reference gate electrode and a sensing gate electrode of 600 × 600 µm^2^, separated by a gap of 185 µm. The Vg supply is connected to the reference electrode and the sensing electrode is connected to the gate terminal of the MOSFET.

### 2.2. Sample Preparation and Regeneration

The mercury ISM is composed of 1 wt% mercury ionophore I, plasticizer 2-nitrophenyl octyl ether (2NOE) of 65.65 wt%, 0.35 wt% sodium tetraphenylborate (NaTPB) as added anion, and polymer substrate Poly vinyl chloride (PVC) of 33 wt%. The mixed ISM units are diluted in 0.003 L of tetrohydrofuran (THF), an organic media [17]. The prepared membrane is stored in a sealed container. Phosphate buffer saline(PBS) is used as the standard solution for preparing the target Hg^2+^ ions. A concentration of 0.02X PBS is normally used. The environmental water sources are checked using ICP-MS prior to testing using the extended gate Hg-ISMFET. To immobilize on the Hg-ISMFET sensor array, 0.3 µL of membrane prepared is dropped, covering both the sensing regions in the sensing gate electrode and the reference gate electrode. The device is then kept at room temperature for 24 hours in a ventilated environment, allowing the organic solvents to evaporate out. The sensor after testing is cleaned and kept in the standard solution for several hours (1–5) for regeneration purposes. The electrical test results can ensure the baseline after regeneration and can also be tested for mercury detection. 

### 2.3. Sensor Measurement

To measure the characteristics of the sensor, a portable biosensor system is utilized. The biosensor system consists of a microcontroller unit, signal acquisition, read out circuitry and USB interface connected to a computer as shown in Figure 1c. The MOSFET used in this study is N-Channel Depletion-Mode DMOS FET (LND150, Supertex Inc., San Jose, CA, USA). The MOSFET is designed to operate in the linear region. It is suitable for amplification applications and has good thermal characteristics. There are two modes of operation based on the user interface software - single mode and burst mode. Measurement data of a single one is collected in single mode operation, while multiple, repeated measurement data are attained in burst mode operation. Throughout the study, burst mode of operation has been adopted. A switch is used to test each of the sensing electrodes. At the reference electrode, a short time pulsed gate voltage with an amplitude of 1 V and width 100 µs is administrated as the gate bias. A drain-source voltage of 2 V is applied throughout the process.

## 3. Results and Discussion

The electrical characteristics of extended gate Hg-ISMFET is shown in Figure 1d. In the absence of a solution, the electrical conduction is through the bulk membrane on application of gate voltage, which causes more potential to drop in the membrane, thus resulting in a low current gain. When the solution is dropped on the ISM, the characteristics of electrical results are modified (Figure 1d). Mobility of the organic charged molecules at the interface are increased when in contact with the solution thereby enhancing the conductivity and thus the drain current. A high current gain is the result of an increased potential drop at the gate dielectric, whereas the potential drops less across the membrane at the interface. So solvation helps mobility and thus the current gain. The ion selective membrane is a polymeric form of organic charged molecules. When gate bias is applied, capacitive response is induced in the interfaces, leading to a potential drop across the dielectric capacitance Cd and hence a change in the drain current of FET. Figure 1e shows the purely capacitive response of the extended gate Hg-ISMFET when gate bias is applied in the presence of a test solution (10^−8^ M mercury ions). To verify that the sensor response is purely capacitive, we test the gate leakage current which is depicted in Figure 1e. In the absence of redox reactions, the gate current Ig quickly relaxes back to almost zero after pulse bias application, indicating that the sensor can be modeled with a purely capacitive response.

The sensor signal called current gain is characterized as the difference in transistor drain current concurrent to the gate bias application. The gain is not definite but is comparable to the trans-conductance gain delineated in case of the conventional FET. As illustrated in Figure 1d the current gain is differentiable in air and in PBS, thus explaining the transition. Accordingly, it offers a superior stability and a stable baseline for the sensor.

**Figure 2:**
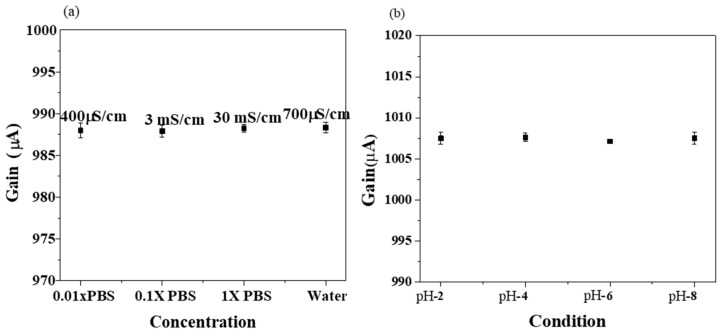
Characteristics of Mercury ion selective ISMFET (Hg-ISMFET). (**a**) Current gain signal of Hg-ISMFET for differing conductivity of test sample. (**b**) Current gain signal of Hg-ISMFET for differing pH of a test sample.

In strong electrolytes, the ion concentration is considered to be proportional to the conductivity of the solution. Conductivity is obtained by measuring the AC resistance of the solution between two electrodes since the conductivity is defined as the reciprocal of resistance when current is passing through a 1 cm^2^ cross-sectional area with a length of 1 cm, where is the unit is µS/cm. For comparison, several test solutions selected to investigate the sensor response towards each of them, which are; 0.01X PBS, 0.1X PBS, 1X PBS and tap water. The conductivity is measured by a conductivity meter B150 from EUTECH. The results are; 400 µS/cm for 0.01X PBS, 3 mS/cm for 0.1X PBS, 30 mS/cm for 1X PBS and 700 µS/cm for tap water. These test solutions were later tested using extended gate Hg-ISMFET (Figure 2a) and the results showed a steady response to the varying conductivity values over a wide region, eliminating the issue of a specific conductivity value while testing different water samples.

Test solutions with pH values ranging from pH-2 to pH-8 were prepared in 0.02X PBS and tested using Hg-ISMFET at a Vg of 1 V. A steady gain response was observed which eliminated the issue of pH selection as shown in Figure 2b. Being independent of the pH and conductivity of the sample, this technique allows real time testing in environmental water samples including drinking water (potable, treated water), tap water, natural water sources such as rivers and lakes, food, beverages, bodily fluids etc. and also provides a fast response time.

### 3.1. Sensing Characteristics of Extended Gate Hg-ISMFET Sensor

**Figure 3:**
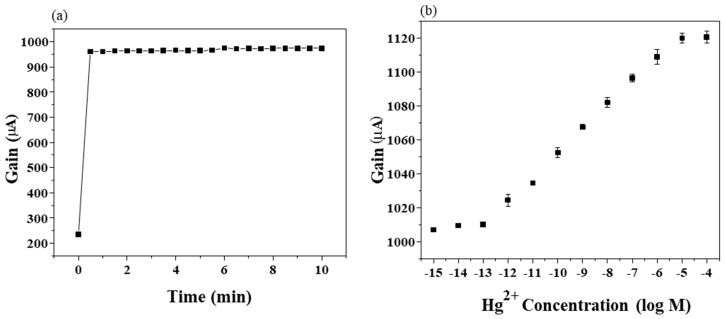
Sensor response; (**a**) Current gain response of extended gate Hg-ISMFET through time. (**b**) Current gain average verses different concentration of Hg^2+^ prepared in 0.02X PBS by extended gate Hg-ISMFET (error bars obtained from multiple tests with *n* = 3).

The device characteristics of extended gate Hg-ISMFET with ISM immobilized on sensing regions is depicted in Figure 3a. Initial measurement in PBS shows an increase in the signal or gain as ISM containing Hg ionophores comes in contact with the test solution and gets stable within few minutes on the application of gate bias. The gain-time curve is shown in Figure 3a. The transition of extended gate Hg-ISMFET sensor response from air to liquid can thus be explained. When liquid comes in contact with the ISM, proper wetting leads to increase in conductivity at the interface and potential drops more at the gate dielectric than in the membrane. On contact with the test solution, the dynamic current gain response gets stable within 5 minutes, i.e. the response time of the extended gate Hg-ISMFET sensor. In Figure 3b, a highly sensitive Hg sensor with an ultralow detection limit of 10^−13^ M and a dynamic range from 10^−13^ M to 10^−5^ M is demonstrated. By analyzing the graph, change in gain is observed at 10^−13^ M, showing an increasing trend with an increase in mercury concentration.

When a fixed V_g0_ is applied, increasing mercury concentration causes an increase in the effective gate voltage due to an increase in the electrical potential of ISM, E_ism_. The E_ism_ value caused by the mercury ions can be calculated by eliminating the V_g0_ applied (1 V). Comparison of E_ism_ with the ideal E_ism,ideal_ from the Nernst equation can be done:

(1) $$E_{ism,\;ideal}=\;c\;+\;\frac{0.05916}{Z}\;\log\;A$$

where *c* is the standard potential, *z* is the valence and *A* is the concentration of target ion. The slope of Vg vs concentration should always ideally follow the Nernst slope from the equation mentioned above, which is $\frac{0.05916}{Z}$. Since in our case for mercury *z* = 2, the slope should be 29.58$\;\frac{mV}{\log M}$. Figure 3b shows excellent detection of mercury with an appreciable sensitivity.

**Figure 4:**
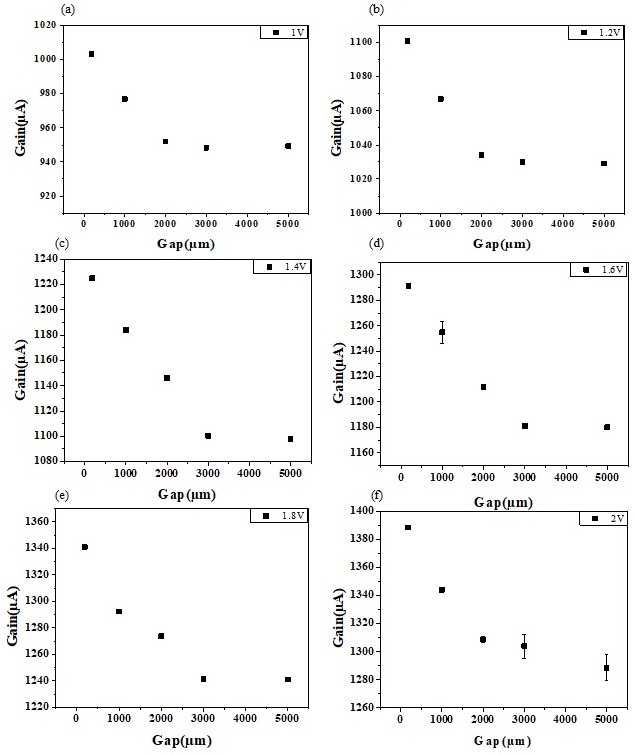
Effect of gap distance between sensing and reference electrodes and applied Vg on current gain. (**a**)–(**f**) Current gain versus different gap distance for fixed Vg.

A systematic study to investigate the sensitivity is carried out by investigating the effect of changing field by means of varying the gap between sensing and reference electrode. With variations in the gap between electrodes, different current signal values were obtained as illustrated in Figure 4. Devices with increasing gap distance (in plane) ranging from 185 µm to 5000 µm were made. For each gap distance, the current gain value in buffer with different Vg in the range of 1 V to 2 V was measured. As the gap distance increases, a decrease in gain was observed, eventually getting stable after a certain gap. Based on the gap distance, two dependent current responses are obtained. The linear region and the saturated region can be distinguished from the figure. The region from 3 mm where no particular change in the gain is visible is the saturation region (low field) with a constant electric field reaching the bulk solution. The region until 3 mm where the decrease in gain occurred is the linear region or the high field region where the gain varies with regards to the gap in a linear manner. In Figure 4a–f, the gate bias application on the range of 1 V to 2 V, for varying gaps of 185, 1000, 2000, 3000 and 5000 µm. The current gain saturates at smaller gaps, i.e., about 2 mm for lower gate voltages. However at higher gaps and at a higher gate voltage, saturation of current gain is shifted further. This implies the dependency of current gain on the real gate voltage and the distance of gap separating the sensing and reference electrodes. Thus, modulation can be done on the extended gate Hg-ISMFET by applying an electric field, operated under a linear region and a larger current gain can be attained.

**Figure 5:**
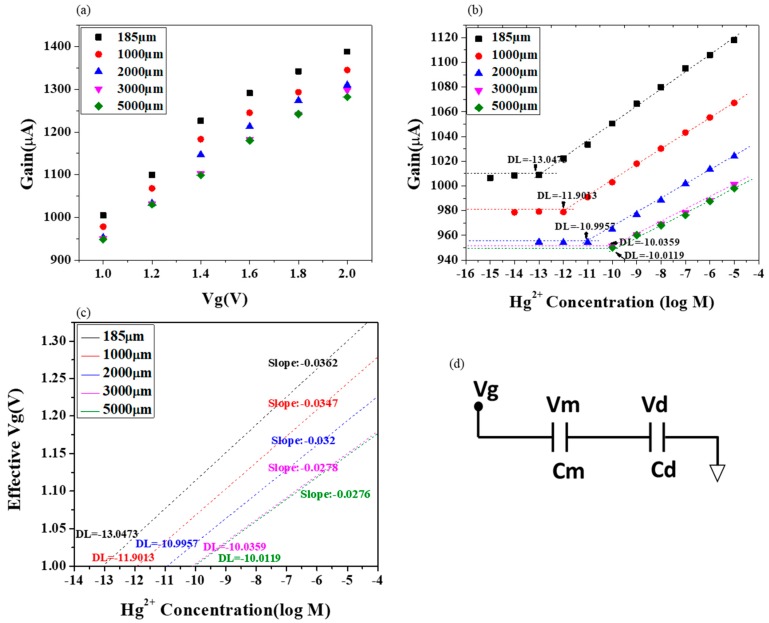
Mercury ion detection using extended gate Hg-ISMFET and sensitivity comparison. (**a**) Gain of Hg-ISMFET versus gate voltage (**b**) Gain of Hg-ISMFET versus log mercury ion concentration. (**c**) Effective gate voltage obtained with respect to log mercury ion concentration. (**d**) Schematic representation of the capacitive model of the sensor.

Its innate to correlate the current change in the signal response of Hg-ISMFET as a function of the gate voltage Vg, for the varying gap distances separating the sensing electrode and the reference electrode as illustrated in Figure 5a. It illustrates the slope of the gain versus Vg graph or the current response of the sensor for a different gap distance, the slope of 3000 µm and 5000 µm are seen to be overlapped or are nearly constant (saturation region of operation) while that of 185 µm, 1000 µm and 2000 µm (linear region of operation) have an increase as the decreasing gap distance. The increase in sensitivity of the Hg-ISMFET sensor can be attributed to its operation in the high field (linear) region. By operating the sensor under linear region a high sensitivity can be achieved, whereas under a low field (saturation) region the sensitivity is lowered and is unallied to the employed strength of an electric field. In consideration with conventional ISE or ISMFET, the electrode used as reference is mostly kept grounded, maintaining a large distance from the FET and a short bias is applied such that the strength of electric field is intensely low between the FET and the reference electrode. Accordingly, the prevailing methods are operated in the saturation region (ISE or ISM-FET), with sensitivity lower and not dependent of electric field applied [23,24,25,26,27,28,29,30,31,32]. Thus, a quantitative comparison of sensitivity of our Hg-ISMFET and the conventional methods can be carried out, developing methods to augment the sensitivity and to advance the detection limit of mercury.

In order to have a thorough perspective of the relation to the improvement in sensitivity when in linear region and to the gap distance a systematic and quantitative study was carried out. Different concentrations of mercury ions ranging from 10^−15^ M to 10^−5^ M were tested in each of the devices with gaps 185 µm, 1000 µm, 2000 µm, 3000 µm and 5000 µm. A gate voltage (Vg) of 1V is applied and the process was carried out from a low concentration to a high concentration (10^−15^ M to 10^−5^ M) of Hg^2+^ ions prepared in 0.02X PBS. The result is shown in Figure 5b. The figure shows that as the gap distance is smaller (185 µm to 2 mm), the slope i.e. the sensitivity of Hg-ISMFET is exigently higher and the detection limit is enhanced by three orders while operating in the linear region. This exemplifies the scope of Hg-ISMFET sensor, in detecting mercury contamination even for a small amount while operating in high electric field in the linear region. 

Comparing quantitatively with the existing methods requires considering the Nernst equation;

(2) $$E_{ISM}=c+\frac{RT}{nF}\log A$$

where *A* is analyte concentration, *R*—gas constant, *T*—temperature and *F*—Faraday’s constant and *n*—the valence of the target ion. For mercury, *n* = 2 

So according to the ideal Nernst equation:

(3) $$E_{ISM,ideal}=c+0.02958\log[Hg^{2+}]$$

Consequently, the ideal Nernstian sensitivity is found to be −29.58 mV/decade mercury ion concentrations. Several research batches have indicated using ISE or ISMFET sensors improving the sensitivity near to the ideal Nernst. Different methods were used to augment the roughness of surface of ISM, thereby increasing the pores and binding sites. Binding can thus be improved, providing a near ideal Nernst sensitivity.

Nevertheless, some researchers have reported a higher than ideal Nernst response without a systematic study to formalize the claim of elevated sensitivity [26,27,28,29,30,31,32].

In Figure 5c, the slopes and detection limits of are mentioned to the sensor calibration curve. The sensor signal in gain vs V_g_ (Figure 5a) and gain vs [Hg^2+^] (Figure 5b) graphs are joined to methodically collect the sensor signal in the Vg format expressed as a [Hg^2+^] function (Figure 5c). The sensor signals at different gap distances of 185 µm, 1000 µm, 2000 µm, 3000 µm and 5000 µm are mathematically fitted to produce their slopes, representing sensitivity. When the operation is in the saturation area (gap = 5 mm) the slope is −27.6 mV/decade [Hg^2+^] closer to the slope of an ideal Nernst.

Operating in linear region (high field region) (gap = 185 µm or 1 mm) the slopes obtained are −36.2 and −34.7 mV/decade [Hg^2+^]. These slopes are higher than the ideal Nernst sensitivity by a fair amount. Hence, we have shown that conventional ISE or ISMFET devices operating in the low field region are limited by the ideal Nernst response. Contrarily, the gating mechanism applied in our extended gate Hg-ISMFET provides a higher sensitivity than the ideal Nernst (−36.2 mV/decade [Hg^2+^]) and facilitates an improved detection limit (10^−13^ M). While operating the device in the high field region, the gain or signal depends upon the gate voltage applied. It also is dependent on the gap distance between the sensing and reference electrodes.

The operation of the device in the linear region, the current signal change being dependent on the supplied Vg and the gap separating the sensing and reference electrodes explains the firm dependence of the response from the sensor and on the strength of field in sample liquid as:

(4) $$E_{Hg-ISMFET}=c+0.02958\eta \log[Hg^{2+}]$$

where *η* is dependent on the gate electrode voltage Vg and on the separating distance of gap in between the gate electrodes, in linear region also called the high field region, *η* > 1 and *η* = 1 in the saturation region. Thus, the mechanism of sensing of Hg-ISM-FET can be explained in a quantitative manner and relate to the limited response of conventional ISE or ISM-FET to the ideal Nernst slope.

### 3.2. Sensor Model of Extended Gate Hg-ISMFET Sensor

In the previous section, it is demonstrated that the extended gate Hg-ISMFET has a lower detection limit than the benchtop instruments, and also has a higher sensitivity than the Nernst behavior. To explain the phenomenon, the relationship between the sensor sensitivity and gap distance between the sensing electrode and the reference electrode is investigated. Most traditional ISE or ISMFET have not considered the relation between sensitivity and the gap distance between the sensing electrodes. The model of traditional ISFET is based on the Helmholtz and Gouy-Chapman model, which uses a non-linear Poisson equation to describe the surface charge density at planar surfaces. A detailed explanation of the difference in principle of our sensor system and traditional ISFET has been described in our previous works [12]. Briefly, the Gouy-Chapman model leads to the Grahame equation, which describes charge density as being dependent on the surface potential. However, in the present work, through our experiments using different gaps between the reference electrode and extended gate, we demonstrate that the charge density is also dependent on the gap and not merely on the surface potential. The traditional methods such as ISFET or ISM-FET operate in the low field region where the electrode used as the reference is kept in a far distance such that the electric field in the bulk solution is the same. Comparing the conditions of bias of our sensor to electrophoresis, larger than 1 V/cm of electric field application between the electrodes [33,34]. With a shorter gap of 185 µm, and applying Vg of 1 V, the field is about ~54 V/cm. Sensitivity of the sensor is prone to the charge screening effect which is caused by extremely short Debye length in high ionic strength solutions. In our device, the gap distance was shortened to 185µm, and a Vg of 1 V was applied and is operated in a high electric field which can modulate the sensor signal and also cause a significant effect to the sensitivity [12]. Usually in conventional ISE, redox reactions occur. It is verified that our sensor response is non-faradaic in nature (Figure 1d). The specific ionophore in the membrane captures the target heavy metal, and the drain current response is thus a function of the potential drops; solution potential drop $\Delta V_{s}$, membrane solution drop $\Delta V_{m}$ and dielectric potential drop $\Delta V_{d}$. In our structure, where ISM is immobilized on both the sensing and reference electrode and the test solution dropped on it, a positive gate voltage applied on the reference electrode, the ISM acts as dielectric and a two plate capacitor is formed and the mobile charges polarizes and form a double layer like structure, forming a series combination of the solution and membrane capacitance, thereby modulating the potential difference at the dielectric and thus the dielectric capacitance. When mercury ions in the test solution binds with the specific ionophore in the ISM, the Cd is altered and as the mercury ion concentration in the test solution increases, the potential drop and hence the transistor current is also increased. Thus the drain current response is a function of the concentration of the target heavy metal ion. This can provide a quantitative and sensitive detection of the mercury concentration.

During the measurement the applied gate bias might not get transduced completely and transform to *g_m_*. The induced separating charges are thus encountered by the potential developed by the diffusion of target ions at the solution-ISM interface. A schematic representation of the capacitances and potential drops in the sensor system is depicted in Figure 5d. According to the two plate capacitor theory;

(5) $$\Delta V_{m}\;=\frac{C_{d}}{C_{m}+C_{d}}\times V_{g}$$

(6) $$\Delta V_{d}=\frac{C_{m}}{C_{m}+C_{d}}\times V_{g}$$

The dielectric potential drop depends only on the membrane capacitance and dielectric capacitance. The solution capacitance Cs can be considered as a function of Cm, because of the structure in which the ISM covers both the sensing and reference electrodes. Membrane capacitance Cm dominates over solution capacitance and hence impacts on the sensitivity. Consequently, an elevated sensitivity than the ideal Nernst can be achieved by regulating the bias constraints and the gap between sensing and reference electrodes. Also detection limits lower than the limits of benchtop laboratory equipment such as ICP-MS can be attained.

### 3.3. Selectivity Characteristics of Extended Gate Hg-ISMFET Sensor

**Figure 6:**
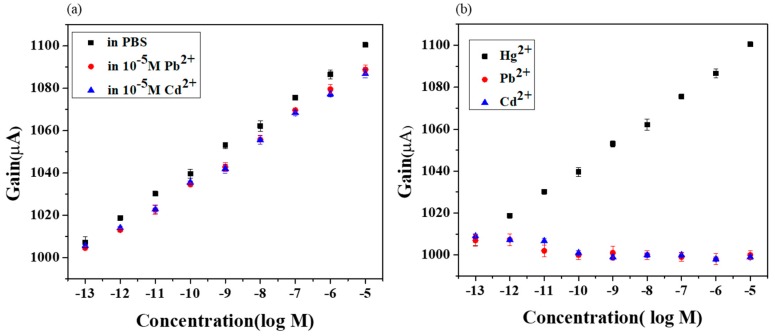
Selectivity characteristics of extended gate Hg-ISMFET sensor measured at 1 V Vg. (**a**) Gain versus heavy metal ion concentration graph of Hg-ISMFET, using fixed interference method. (**b**) Gain versus heavy metal ion concentration graph of Hg-ISMFET, using separate solution method (error bars obtained from multiple tests with *n* = 3).

The selectivity behavior is of paramount importance in determining the characteristics of the extended gate Hg-ISMFET. The influence of interfering ions is described in terms of selectivity coefficients. To assess the selectivity characteristics, IUPAC recommends two methods: the fixed interference method (FIM) and the method of separate solution (SSM) [35]. The selectivity characteristics are illustrated in Figure 6. It demonstrates the outcomes from both FIM and SSM, respectively. Figure 6a shows the FIM results which demonstrate sensitivity. The range of concentrations of mercury used as primary ion were 10^−13^ M to 10^−5^ M, and were prepared in a fixed 10^−5^ M Pb(NO_3_)_2_ and 10^−5^ M Cd(NO_3_)_2_ concentrations. The sensitivity of the extended gate Hg-ISMFET remained comparable. However, there was a very slight baseline signal shift. SSM is used to demonstrate the selectivity. For SSM, lead and cadmium solution were prepared in 0.02X PBS ranging from 10^−13^ M to 10^−5^ M. The experiment was carried out by testing lead ions from low concentration to high concentration (10^−13^ M–10^−5^ M) followed by Cadmium ions from low concentration to high concentration (10^−13^ M–10^−5^ M) and finally testing mercury ions in the range of 10^−13^ M to 10^−5^ M (Figure 6b). Under high interfering ion concentrations, detection limit and sensitivity was not compromised. This allows detection of target ions in environmental water samples, food, beverages, industrial wastewater and medicines. This performance implies that our sensor is highly selective towards mercury and has less affinity towards other ions. Also it showcases that the device maintained the dynamic range and detection limit while in the presence of other ions. Further studies and improvements are possible by modifying it to a sensor array configuration, where each of the sensors can detect various heavy metals. As a result, a more accurate and easy detection of multiple heavy metal ions can be done.

Previous results (Figure 2) show the independent nature of extended gate Hg-ISMFET. Hence, water samples can be directly tested without filtering or pre-treating, and the sensor would not need frequent re-calibrations. This methodology can thus provide rapid detection of target heavy metal ions, at a very low cost which is also consumer friendly.

The issue of selectivity has always been a drawback of traditional methods like ISE. From experimental results, some interfering ions are found to impose a strong influence on sensitivity towards target ion, whereby limiting the detection to higher values [36]. Several studies were done to increase the selectivity of the particular target ion in the presence of several high concentration interfering ions [37]. Most of them faced the problem of a decrease in sensitivity while in the presence of other interfering ions but showed significant selectivity characteristics towards the target ion while showing no obvious response to the interfering ions [37,38]. The highly selective characteristics can be described by site binding theory, according to which the competition between interfering ions and target ion to bind onto the ISM is explained. The present extended gate Hg-ISMFET reveals high selectivity toward Hg^2+^ ion versus other cations (Figure 6). Common interfering ions, Cd^2+^ and Pb^2+^ have no apparent effect on the functioning of this ISMFET.

Being highly selective towards the target heavy metal ion, even in the presence of high concentrations of interfering ions, it opens up possibilities in testing in various other water based systems like natural water sources, tap water, drinking water, food, beverages and so on. Previously some studies were carried out for detecting mercury in environmental water samples and results with a good sensitivity were also obtained [38]. However dilution and pre-treatment needs to be done, thus making the process difficult and uneasy for real time testing. Other factors like conductivity and pH also needed to be taken care of while testing environmental samples.

#### Real Time Testing by Extended Gate Hg-ISMFET in Industrial Waste Water Samples

Real time testing (blind test) was conducted in industrial waste water to determine the concentration of mercury and compared with ICP-MS results, thereby validating our sensor and its capability to do real time testing in water based samples. Six samples of industrial waste water were collected and tests were performed using an extended gate Hg-ISMFET sensor. The results were confirmed further through comparison with ICP-MS results. Initially, the device was calibrated using mercury diluted in a standard solution i.e. 0.02X PBS and the calibration curve was used to extrapolate the blind test result value. Six of the samples were randomly tested and the data was extrapolated using the original calibration curve. Later the test results were compared with the ICP-MS results and the efficiency of the extended gate Hg-ISMFET was validated. Since our sensor has a lower detection limit than ICP-MS, we were able to detect the lower concentration values which were unable to be traced by ICP-MS. As shown in Table 1, the comparison of both the results and shown and sample F shows the lowest concentration which was traced by our sensor but was unable to be detected by the benchtop instrument. We were able to acquire the results for mercury as in the same order as of the results from ICP-MS. Our sensor and ICP-MS operate on different methodologies, and therefore the blind test results within the same order as the ICP-MS results can be considered sufficient. However further studies and improvements are possible by modifying the sensor to a sensor array configuration, where each of the sensors can detect various heavy metals. Thereby more accurate and easy detection of multiple heavy metal ions can be done. A comparison of our work with other reported FET based mercury ion sensors is summarized in Table 2. 

## 4. Conclusions

In this research, ion selective membrane coated extended gate FET sensors are being used to manifest a novel methodology which outweighs the ideal Nernst sensitivity limit while providing an elevated sensitivity. The sensitivity increment staggeringly curtails the detection limit of mercury ion concentrations magnitude to several orders. Systematic investigation was carried out by measuring peculiar and different sensor design arrangements and bias voltage, revealing that in a high field domain, the limit of the ideal Nernst sensitivity can be outweighed. By achieving a sensitivity of −36 mV/lob [Hg^2+^] with our design we can surpass the ideal Nernst slope restriction (−29.58 mV/lob [Hg^2+^]) with a wide dynamic range of detection (10^−13^ to 10^−5^ M Hg^2+^. Comparing the result with ICP-MS, having a limit of detection 10^−11^ M, we were able to achieve a detection limit of 10^−13^ M which is two orders lower. This result is an improvement over other laboratory based equipment, which only provides a DOP of 10^−8^ M, is quite expensive and is inconvenient to implement. Having an extended gate design also reduces the stability issue since a single FET is being used for an array of sensors. Selectivity characteristics of extended gate Hg-ISMFET reveal that high concentrations of interfering ions do not cause axiomatic changes in the sensitivity and detection limit. The results are crucial, providing hassle less testing of various environmental water samples without pre-treatments and re-calibrations. Integration of this refined sensing mechanism with suitable modifications can provide an impeccable way to conduct expeditious screening of heavy metal ion contamination in real time. An integrated chip with an array of sensors having distinct ionophores can collectively detect various analytes. Since the developed technology costs a fraction of the conventional models, it extends the possibilities for utilizing commercialized water quality monitoring devices.

## Figures and Tables

**Table 1:** Comparison of results obtained from ICP-MS and extended gate Hg-ISMFET.

Sample No.	Extended Gate Hg-ISMFET	ICP-MS
A	1.36 × 10^−11^ M	1.9931 × 10^−11^ M
B	7.72 × 10^−11^ M	2.850 × 10^−11^ M
C	4.10 × 10^−11^ M	3.1327 × 10^−11^ M
D	1.86 × 10^−11^ M	1.7677 × 10^−11^ M
E	2.55 × 10^−11^ M	2.613 × 10^−11^ M
F	1.583 × 10^−13^ M	ND

**Table 2:** Comparison of contemporary FET based mercury sensors

Methodology	Dynamic Range	Detection Limit	Response Time	Features
MoS_2_ nanosheet/gold nanoparticle hybrid field-effect transistor (FET) sensor [37]	10^−10^–10^−8^ M	10^−10^ M	1–2 s	DNA as a ion selective agent; functionalization of FET with DNA required
Organic polymer field-effect transistor [39]	10^−6^–10^−3^ M	10^−6^ M	200 s	Functionalization with DNA required; extended stability in marine environment demonstrated
Reduced graphene oxide field-effect transistor [40]	10^−9^–10^−6^ M	10^−9^ M	Several seconds	Functionalization with DNA required
Micropatterned reduced graphene oxide FET [41]	10^−9^–10^−10^ M	10^−9^ M	50 s	Functionalization with protein required
Extended gate organic FET [42]	10^−11^–10^−5^ M	10^−11^ M	--	Functionalization with dipicolylamine required
This work	10^−13^–10^−5^ M	10^−13^ M	5 min	Ion selective polymer membrane is used as receptor
